# Pan LF-ELISA using *Bm*R1 and *Bm*SXP recombinant antigens for detection of lymphatic filariasis

**DOI:** 10.1186/1475-2883-6-10

**Published:** 2007-10-26

**Authors:** Rohana Abdul Rahman, Cheah Hwen-Yee, Rahmah Noordin

**Affiliations:** 1 Institute for Research in Molecular Medicine (INFORMM), Suite 110, Eureka Complex, Universiti Sains Malaysia, 11800 Penang, Malaysia

## Abstract

**Background:**

Anti-filarial IgG4 antibody has been shown to be a good marker for detection of lymphatic filaria infection. Previous studies demonstrated that anti-filarial IgG4 assay using *Bm*R1 recombinant antigen was highly specific and sensitive for detection of brugian filariasis. For bancroftian filariasis, an equivalent assay employing recombinant antigen expressed from the ORF of *SXP1 *gene has been reported. In order to detect infections by all species of lymphatic filarial, *Bm*R1 and *Bm*SXP recombinant antigens were employed in the development of a pan LF-ELISA.

**Methods:**

*Bm*R1 was previously produced while *Bm*SXP recombinant antigen was produced by cloning the ORF of *SXP1 *gene from a *Brugia malayi *cDNA library, followed by expression in a bacterial expression system. Subsequently, each of the purified recombinant antigens (*Bm*R1 and *Bm*SXP) and mixture of different ratios of the two antigens (1:1, 2:1 and 1:2) were tested using IgG4-ELISA with various categories of infection and normal human serum samples.

**Results:**

The results showed that both recombinant antigens were highly specific (99%–100%). For detection of brugian filariasis, *Bm*R1 antigen alone and the mixture of *Bm*R1 with *Bm*SXP (1:1) gave 98% sensitivity; while *Bm*SXP antigen alone showed 84% sensitivity. For detection of bancroftian filariasis, *Bm*SXP antigen was more sensitive (95%) than assays using either *Bm*R1 or mixtures of the two recombinant antigens.

**Conclusion:**

A sensitive and specific pan LF-ELISA for detection of lymphatic filariasis was successfully developed using two adjacent wells, each separately coated with *Bm*R1 and *Bm*SXP.

## Background

Human lymphatic filariasis (LF) is caused by three species of tissue dwelling filaria nematodes namely *Wuchereria bancrofti, Brugia malayi *and *Brugia timori*. An estimated 120 million people worldwide are affected by these infections [1]. Lymphatic filariasis causes a spectrum of clinical and sub-clinical manifestations which include recurrent fever, adenolymphangitis, renal and lymphatic damage, chyluria, hydrocoele and elephantiasis. A WHO initiated Global Program for Elimination of Lymphatic Filariasis (GPELF) is ongoing and the target year is 2020 [2].

Diagnostic methods for brugian and bancroftian filariasis include night blood examination, immunoassays, PCR, ultrasound and lymphoscintigraphy. For brugian filariasis, the immunoassay available include Brugia Rapid, an immunochromatography IgG4 antibody detection test [3]. The rapid test is based on *Bm*R1 recombinant antigen expressed from *Bm17DIII *gene [GenBank: AF225296] and has been shown to be highly specific and sensitive for the detection of *B. malayi *and *B. timori *[3-6].

The ORF of *SXP1 *gene was identified by immunoscreening of *B. malayi *cDNA library with immune sera from microfilaria positive patients with brugian and brancroftian filariasis [7]. The recombinant protein derived from the gene has been employed in the identification of 83% (64/72) to 100% (72/72) of bancroftian filariasis patients when tested with IgG4-ELISA [8]. A rapid flow-through IgG immunofiltration test using *Wb*SXP-1 recombinant antigen was shown to detect 39% of sera from *B. malayi *microfilaraemic individuals, however it demonstrated a much higher sensitivity (91%; 30/33) for detection of *W. bancrofti *infection [5]. In another study it was shown to be able to identify 90.8% (N = 70) and 91.4% (N = 140) of *B. malayi *and *W. bancrofti *microfilaria carriers respectively [9].

The availability of a sensitive and specific assay that can detect all species of lymphatic filariasis would be advantageous in areas with mixed infections and for screening of foreign workers from brugian and bancroftian filariasis endemic countries. In our effort to develop such an assay, we employed *Bm*R1 (previously produced) and a recombinant antigen derived from cloning and expression of the ORF of *SXP1 *gene, in the development of an a pan LF-ELISA that detects all species of lymphatic filariasis.

## Methods

### PCR amplification of the ORF of *SXP1 *gene

The sequence of the ORF of *SXP1 *gene (462 bp) was obtained from GenBank [accession no: M98813]. Amplification of the gene was attempted from two libraries namely *B. malayi *adult worm library (*Bm*RN, previously constructed in our laboratory) and *W. bancrofti *adult female library (from NIAD/NIH Filariasis Research Project Repository Centre (FR3) [10]. The primers employed in PCR amplification were as follows: SXP1-F (5' GTC ACT TCA TCA CTC AAT 3') and SXP1-R (5' CTA TTT ATT ACT TTT TGT CG 3').

### Cloning of *BmSXP1*

The amplicons were ligated into TOPO TA cloning vector (pCR^®^2.1-TOPO, Invitrogen, USA) and then transformed into *Escherichia coli *strain XL1 blue (Stratagene, USA). The DNA sequences of the transformed clones were analyzed with vector NTI software (Invitrogen, USA).

A base mutation at base 104 in the nucleotide sequence (T was changed to C) was repaired by *in-vitro *site directed mutagenesis using a commercially available kit (Stratagene, USA). Subsequently the recombinant plasmid (*BmSXP1*/pTOPO) was digested with *Eco*R1 restriction enzyme, then ligated into *Eco*R1-restricted pPROEX™HTa expression vector (Life technologies, USA) and finally transformed into *E. coli *strain TOP 10.

### Expression and purification of recombinant *BmSXP *protein

The recombinant bacteria was cultured in Terrific broth containing 100 μg/ml ampicillin and incubated at 200 rpm, 37°C until the optical density reached 0.5. The culture was induced with IPTG (Isopropyl-β-thiogalactopyranoside) at a final concentration of 1 mM and incubated at 30°C in an incubator shaker. Cells were harvested after 6 h of induction and disrupted by sonication at 200 W for 10 min (Misonic 3000, New York) in lysis buffer. This was followed by centrifugation at 12000 × g at 4°C for 30 min and the supernatant was affinity purified using Ni-NTA superflow resin (Qiagen, Germany). The purified protein fractions were pooled, concentrated to 2 mg/ml, aliquoted and stored at -70°C. The same concentration of stock *Bm*R1 recombinant antigen was employed in the IgG4-ELISA described below.

### Western Blot

Approximately 100 μg/ml of *Bm*SXP antigen was subjected to 10% SDS-PAGE for 2 h at 100 V and transferred onto nitrocellulose membrane (Osmonics) using semi-dry transblot (BioRad, USA). The membrane was cut into strips, blocked for 1 h with 1% blocking solution (Roche Diagnostics, Germany), then washed. Subsequently the strips were incubated with serum samples diluted 1: 100 with 0.5% blocking solution, for 3 h at room temperature (rt). After a washing step, the strips were incubated with monoclonal IgG4-HRP (CLB, Switzerland) at 1: 3000 for 1 h, rt. BM chemiluminescence's blotting reagent (Roche Diagnostics, Germany) and X-ray films (Kodak, USA) were employed for development of the blots.

### Human serum samples

A total of 333 serum samples were employed, as shown in Table 1. These were from various serum banks, collected according to the ethical requirements of each institution. The samples were as follows: *W. bancrofti *sera from endemic areas of India and Indonesia; *B. malayi *sera from endemic areas of Malaysia and Indonesia; *B. timori *sera samples from Alor, Indonesia, *Loa-loa *and *Onchocerca volvulus *sera from microfilaraemic individuals. Sera from other parasitic infections, endemic normals and non-endemic normals (healthy blood donor) were from Malaysia.

**Table 1:** Serum samples employed in the study

Type of infection serum	Number of samples
*W.b. *(mf+)	55
*W.b chronic *(CFA+)	5
*W.b acute *(CFA+)	8
*W.b amicrofilaraemic *(CFA+)	9
*Brugia malayi *(mf+)	101
*Brugia timori *(mf+)	10
Strongyloides stercoralis*	10
Other soil-transmitted helminthes (STH)* (single or mixed infections with *Ascaris lumbricoides*, *Trichuris trichiura*, hookworm, *Toxocar*a)	45
Extraintestinal amoebiasis **	10
Normal serum (from non-endemic area)	34
Normal (from endemic area)	16
*Loa-loa *(mf+)	10
*Onchocerca volvulus *(mf+)	20
Total	333

Notes:

1. Circulating filarial antigen positive (CFA+), *Wuchereria bancrofti *(*W.b.*), microfilaria positive (mf +)

2.*Parasite seen in stool samples, except for *Toxocara *infection which was diagnosed based on positive serology with consistent clinical presentation.

3. ** Positive serology with consistent clinical and radiological findings.

4. Serum samples kindly provided by the other scientists were as follows:

i. *W. bancrofti*: from Dr. B. Ravindran from ICMR, Bhubaneswar, India, and Dr. Taniawati Supali from University of Indonesia, Jakarta, Indonesia

ii. *B. malayi *from Indonesia: Dr. Taniawati Supali from University of Indonesia

iii. *L. loa *and *O. volvulus*: Dr. Peter Fischer from Bernhard Nocht Institute for Tropical Medicine, Hamburg, Germany

### IgG4-based ELISA

The procedure employed was as reported previously [11], with optimizations of the concentration of recombinant antigen, dilution of serum samples and secondary antibody conjugate. All washing steps were performed using a microtitre plate washer (Columbus/Columbus Pro Washer, TECAN Austria GmbH) with PBS 0.05% Tween 20 (PBS/T), 5 times at 5 min each. Briefly, a microtitre plate (96 wells, Maxisorp™ Nunc, Denmark) was coated with 100 μl of 20 μg/ml recombinant antigen in 0.02 M bicarbonate buffer, pH 9.6 and incubated overnight at 4°C, followed by 37°C for 2 h and then washed. After blocking and washing step, 100 μl serum sample/well (1:50 in PBS, pH 7.2) was incubated at 37°C for 2 h, washed then followed by 30 min incubation with 100 μl monoclonal anti-human antibody IgG4-HRP(1:4500 in PBS ; CLB, Netherlands). Following a final wash, the color development was accomplished using ABTS substrate (Roche Diagnostics, Germany) for 30 min at 37°C. The plates were read at 410 nm test filter and 490 nm reference filter using an ELISA plate reader (DynaTech MR 5000, USA). All sera were tested in duplicate and results were expressed as optical density (OD) values. An OD of ≥ 0.3 was employed as the cut-off value for positive case, this was derived from the mean OD plus three standard deviations after testing 50 serum samples of normal individuals from a filariasis endemic area in Malaysia.

### Pan LF-ELISA using *BmR1 *and *BmSXP*

In the phase I of the study, each of the purified recombinant antigens (*Bm*R1 and *Bm*SXP) and mixtures of different ratios of the two antigen (1:1, 2:1, 1:2) were tested using IgG4 ELISA and 50 serum samples from each of the following categories: (1) individuals with *B. malayi *microfilaria; (2) individuals with *W. bancrofti *microfilaria; (3) patients with soil transmitted helminthes (STH) and (4) normal individuals from filarial non-endemic area (healthy blood donors from Malaysia). Subsequently in the phase II of the study, the 'shortlisted antigens' namely *Bm*R1, *Bm*SXP and a mixture of equal proportions of the two antigens were tested with the rest of the serum samples (N = 133).

## Results

### Cloning and Expression of the ORF of *SXP1 *gene

The ORF of *SXP1 *gene was successfully amplified from cDNA library of *Bm*RN, however several attempts at amplifying the gene from *W. bancrofti *female library were unsuccessful. The mutation observed at base 104 (from the start codon of the gene) in the *SXP1*/pTOPO-TA was successfully corrected. After subcloning into pPROEX™HTa expression vector, the length of the nucleotides from start codon of the vector to the stop codon of the gene is 585 bp. The expression of the recombinant gene produced a soluble protein and the yield of the purified *Bm*SXP recombinant protein obtained was approximately 5 mg/L of culture.

### Western Blot

**Figure 1:**
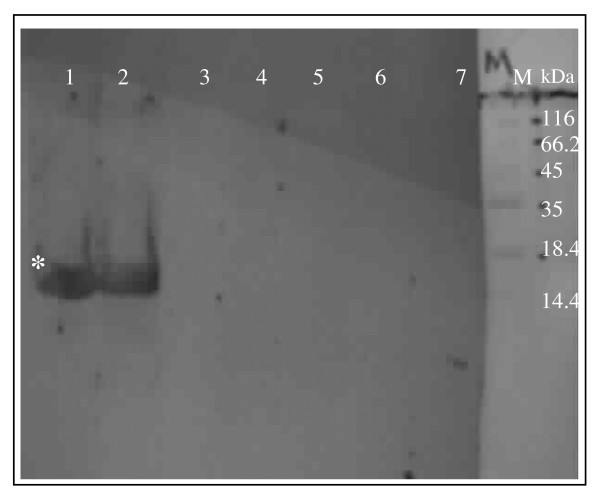
**Western blot results of *BmSXP *recombinant antigen probed with various serum samples**. The following serum samples were employed as primary antibodies: Lane 1: Serum from a *W. bancrofti *mf+ patient (India). 2: Serum from a *W. bancrofti *mf+ patient (Indonesia). 3: Serum from an ameobiasis patient. 4: Serum from a patient with *Ascaris lumbricoides*. 5: Serum from a patient with *Trichuris trichiura*. 6: Serum from a normal individual from filaria endemic area. 7: Serum from a normal individual from a non-endemic area. M: Low molecular weight marker (Fermentas). *: ~24.7 kDa *Bm*SXP recombinant protein

As shown in the western blot analysis in Figure 1, *Bm*SXP recombinant protein with a size of approximately 24.7 kDa was specifically recognized by *W. bancrofti *infection sera. No cross reactivity was observed with serum samples from soil-transmitted helminthes or with normal sera.

### IgG4-ELISA using *BmR1 *and *BmSXP*

The results of the phase I of the study showed that for detection of brugian filariasis, *Bm*R1 antigen alone and mixtures of *Bm*R1 with *Bm*SXP (either 1 *Bm*R1:1 *Bm*SXP or 2 *Bm*R1:1 *Bm*SXP) gave 100% sensitivity while *Bm*SXP antigen alone showed 94% sensitivity (Table 2). For detection of bancroftian filariasis, *Bm*SXP antigen alone and a mixture of the two antigens (either 1 *Bm*R1:1 *Bm*SXP or 1 *Bm*R1:2 *Bm*SXP) were more sensitive (94%) than using *Bm*R1 alone. The 'shortlisted' antigen candidates selected for best sensitivity and specificity for detection of brugian and bancroftian filariasis were thus *Bm*R1, *Bm*SXP, and 1:1 mixture of the two antigens.

**Table 2:** Reactivities of five different antigen preparations to different categories of serum samples in phase I of the study

	**Categories of serum samples**			
**Preparation of antigen**	*Bm *mf+	*Wb *mf+	Serum from STH infections	Normal
*Bm*R1	50/50 (100%)	6/50 (12%)	0/50	0/50
*Bm*SXP	47/50 (94%)	47/50 (94%)	1/50	0/50
Ratio of 1 *Bm*R1:1 *Bm*SXP	50/50 (100%)	46/50 (92%)	1/50	0/50
Ratio of 2 *Bm*R1:1 *Bm*SXP	50/50 (100%)	20/50 (40%)	0/50	0/50
Ratio of 1 *Bm*R1:2 *Bm*SXP	46/50 (92%)	47/50 (94%)	1/50	0/50

Results obtained from phase II of the study using the three shortlisted candidates on the rest of the serum samples are shown in Table 3. Table 4 shows the sensitivity and specificity of the three shortlisted antigens determined from results in Tables 2 and 3. Thus for detection of brugian filariasis, *Bm*R1 antigen alone and a 1:1 mixture of the two antigens gave 98% sensitivity while *Bm*SXP antigen alone showed 84% sensitivity. For detection of bancroftian filariasis, *Bm*SXP antigen alone was more sensitive (95%) than assay using *Bm*R1 alone (14%) or mixture of the two recombinant antigens (84%). With regard to specificity, all antigen preparations gave excellent results i.e. 99–100% specific.

**Table 3:** Reactivities of 'shortlisted' antigens to different categories of serum samples (not inclusive of sera in Table 2) in phase II of the study.

	**Categories of serum samples**				
**Preparation of antigen **(ratio)	*B. malayi *and *B. timori *mf+	*W. bancrofti *mf+ and/CFA+	*L. loa*	*O. volvulus*	Other infections
*Bm*R1	59/61 (97%)	5/27 (19%)	0/10	0/20	0/15
*Bm*SXP 1	46/61 (75%)	26/27 (96%)	7/10	11/20	0/15
*Bm*R1:*Bm*SXP (1:1)	59/61 (97%)	19/27 (70%)			0/15

**Table 4:** Sensitivity and specificity of the various antigens for detection of brugian and bancroftian filariasis. Reactivities to *L. loa *and *O. volvulus *were excluded from the specificity evaluation.

Type of antigen	Sensitivity to *B. malayi *and *B. timori*	Sensitivity to *W. bancrofti*	Specificity
*Bm*R1	109/111 (98%)	11/77 (14%)	0/115 (100%)
*Bm*SXP	93/111 (84%)	73/77 (95%)	1/115 (99%)
*Bm*R1: *Bm*SXP (1:1)	109/111 (98%)	65/77 (84%)	1/115 (99%)

Since both *Bm*R1 and *Bm*SXP seemed to be sensitive (98% and 84% respectively) in detecting anti-filarial IgG4 in brugian filariasis patients, comparison of the degree of reactivities of *Bm*R1 and *Bm*SXP to serum samples from *B. malayi *patients were made by comparing the mean ODs obtained by each of the recombinant antigens using 30 serum from *B. malayi *microfilaraemic patients which reacted with both antigens. As shown in Table 5, t-test analysis demonstrated that mean OD obtained using *Bm*R1 is significantly higher (p < 0.001) than that obtained using *Bm*SXP.

**Table 5:** T-test analysis of the comparison of mean ODs of IgG4 assays on 30 *B. malayi *serum samples using *Bm*R1 and *Bm*SXP recombinant antigens.

		*Bm*SXP	*Bm*R1
Mean		0.891	2.086
Standard deviation		0.602	0.989
Mean standard error		0.110	0.180
Test value = 0	t value	8.116	11.559
	Sig (2-tailed)	p < 0.001	p < 0.001
	Mean difference	0.891	2.086
	95% C.I.	0.667–1.116	1.717–2.455

## Discussion

To ensure success of the Global Program for Elimination of Lymphatic Filariasis (GPELF) by year 2020, diagnostic tools play an important role in mapping of endemic areas, monitoring effectiveness of the mass-drug administration and surveillance post-elimination. Commercially available sensitive and specific antigen and antibody detection tests are available for bancroftian and brugian filariasis respectively. Nevertheless, there is a need for an assay that can detect both bancroftian and brugian filariasis with a high degree of sensitivity and specificity. This will be especially useful for testing in areas with mixed infection or with unknown species of lymphatic filarial, and for screening of foreign workers from filarial endemic countries.

*Bm*RI recombinant protein, which is employed, both in IgG4 ELISA format and in a commercially available rapid immunochromatography test (*BRUGIARAPID*™; Malaysian BioDiagnostic Research Sdn. Bhd.) has been shown to be highly specific and sensitive for detection of *B. malayi *and *B. timori *infections [3,6,11-15]. However, it displayed variable reactivities when tested with sera from *W. bancrofti *infection [4,5]

For bancroftian filariasis, recombinant antigen derived from the ORF of *SXP1 *gene (*Wb*-SXP1) has been shown to be highly sensitive (91–100%) as a diagnostic reagent when employed in IgG4-ELISA [7,8] or in a rapid IgG immunofiltration assay [5,9]. For detection of brugian filariasis, in one study the rapid test was reported to detect 90.8% (N = 70) of *B. malayi *patients [9] while in another study the detection rate for brugian filariasis was 39% [5]. The *B. malayi *homologue of the SXP-1 gene (*Bm*-SXP-1) was shown to successfully identify 83% (64/72) of bancroftian filariasis patients [8] but in another study it was not reactive with 16 sera from patients infected with *B. malayi *[7].

A closely related recombinant antigen, Bm14 (or BmM14), codes for a protein that appears to correspond to a 13 kDa native *B. malayi *antigen [16]. This clone was isolated from *B. malayi *adult male worm cDNA library with sera of bancroftian filariasis patients [7]. Alignment of sequences of BmM14 [GenBank: M95546]and SXP-1 revealed more than 90% similarity. Bm14 recombinant protein was reported to equally reactive (90%, N = 111) in an ELISA using sera from patients with brugian and bancroftian filariasis [16]. In a more recent study, Bm14-IgG4 ELISA was reported to demonstrate sensitivity up to 91% (32/35) for detection of *W. bancrofti *and 96% (27/28) for detection of *B. malayi *infections [5]. The assay has also been employed in longitudinal study of bancroftian filariasis in Egypt, whereby the levels were shown to decrease post-treatment [17,18].

Utilizing the above information, we employed *Bm*R1 recombinant antigen and an antigen derived from cloning of the ORF of *SXP1 *gene in the development of an Ig4-ELISA that can detect antibodies against all species that cause lymphatic filariasis. Although were unable to show that *Bm*SXP and *Wb*SXP recombinant antigens have the same diagnostic potential to detect IgG4 antibodies to *W. bancrofti*, the high level of sensitivity (95%) and specificity (99%) achieved by our *Bm*SXP recombinant antigen for detection of bancroftian filariasis would unlikely be significantly different from that achievable by *Wb*SXP recombinant antigen had we been successful in producing this antigen. This assumption is based on the previous reports of sensitivity of *Wb*SXP for detection of bancroftian filariasis namely 91% (n = 33) [5], 91.4% (n = 140) [9], and 100% (n = 72) [8]. Subsequently *Bm*R1 and *Bm*SXP were employed alone and in various ratios (1 *Bm*R1:1 *Bm*SXP; 1 *Bm*R1:2 *Bm*SXP; 2 *Bm*R1:1 *Bm*SXP) in IgG4-ELISA to determine the best antigen preparation that will enable the most sensitive and specific detection of all three species of lymphatic filaria. Since both *Bm*R1 and *Bm*SXP are soluble antigens and are suspended in the same buffer, no problem arose when the recombinant antigens were mixed.

*BmSXP *was more sensitive (95%) in detecting of *W. bancrofti *infection as compared to *Bm*R1 (14%). On the other hand *Bm*R1 was more sensitive than *Bm*SXP in detecting *B. malayi *infection (98% and 84% respectively). Comparison of the mean ODs obtained from 30 *B. malayi *serum samples showed that *Bm*R1 was significantly more reactive than *Bm*SXP for detection of IgG4 antibodies in brugian filariasis patients' sera. No such comparison was made between degree of reactivities of *Bm*R1 and *Bm*SXP with serum samples from *W. bancrofti *patients, since it was evident that the former antigen was insensitive for use as a diagnostic reagent for bancroftian filariasis.

The results also showed that to achieve the best sensitivity and specificity for detection of both brugian and bancroftian filariasis, two separate wells should be employed, one for each kind of recombinant antigen. Combining the recombinant antigens appeared to decrease the sensitivities for detection of either one of the infection, probably because of the reduced availability of antigen binding sites in the wells as compared to that available when only one kind of recombinant antigen was employed.

With regard to specificity of the antigens, all preparations were very specific (99–100%) when tested with serum samples from other infections (except *L. loa and O. volvulus*) and from normal individuals. Cross-reactivities detected with *L. loa and O. volvulus *samples makes the assay not useful in areas co-endemic with these infections. However this does not reduce the significance of this assay since worldwide there are vast lymphatic filariasis endemic areas outside Africa that are not endemic for non-lymphatic filaria.

Since both recombinant antigens react (to different degrees) with both brugian and bancroftian filariasis infection sera, the assay developed in this study is not useful for species identification. However in the context of GPELF, this is not crucial since the treatment for all species is quite similar.

## Conclusion

A sensitive and specific pan LF-ELISA for detection of lymphatic filariasis was successfully developed using two adjacent wells, each separately coated with *Bm*R1 and *Bm*SXP. The pan LF assay using *Bm*R1 and *Bm*SXP recombinant antigens has been converted to a rapid immunochromatography test and is now undergoing multicentre validation studies.

## Competing interests

The author(s) declare that they have no competing interests.

## Authors' contributions

RN – conceive, design and supervise the study, participated in the laboratory work and analysis of the results, performed major editing of the manuscript

RAR – performed the major part of the laboratory work and analysis of the results, wrote the major part of the first draft of the manuscript

CHY – participated in the laboratory work and analysis of the results, participated in writing the first draft and editing of the manuscript.

All authors read and approved the final manuscript
